# Supplementary material for: Development of social isolation and social network assessment tool for older adults: A Delphi survey
Source: PLoS One. 2025 May 9;20(5):e0323198. doi: 10.1371/journal.pone.0323198 (PMC12064011; doi:10.1371/journal.pone.0323198)
Supplement: S1 Text — The questionnaire contains all items of SISN assessment. (DOCX) [file pone.0323198.s001.docx]

**Social Isolation and Social Network (SISN) Profile**

**Ⅰ. Objective isolation**

**The following questions are about your objective isolation. Please mark 'v' in the appropriate box.**

**1. How many family members or housemates live with you?**

□ None

□ 1 person

□ 2 people

□ 3-4 people

□ 5 or more people

**2. What public transportation is available where you currently live?**

□ None

□ Taxi

□ Bus

□ Subway

□ All available

**3. Please select whether public transportation is conveniently accessible from your home.**

□ Very poor

□ Poor

□ Fair

□ Good

□ Very good

**4. Please indicate how many times you go out of the house on average per day.**

□ Never

□ Once

□ 2-3 times

□ 4-5 times

□ 6 or more times

**5. Do you have acquaintances who visit you every day, and if so, how many?**

□ None

□ 1 person

□ 2 people

□ 3-4 people

□ 5 or more people

**6. How many people call you or ask how you are every day?**

□ None

□ 1 person

□ 2 people

□ 3-4 people

□ 5 or more people

**7. Are you currently communicating through Social Network Sites?**

□ Never

□ Rarely

□ Sometimes

□ Often

□ Very often

**Ⅱ. Subjective isolation**

**The following questions are about your objective isolation. Please mark 'v' in the appropriate box.**

**1. Over the past month, I've really missed my close friend.**

□ Strongly agree

□ Agree

□ Neutral

□ Disagree

□ Strongly disagree

**2. Over the past month, I have felt a general sense of emptiness**

□ Strongly agree

□ Agree

□ Neutral

□ Disagree

□ Strongly disagree

**3. Over the past month, I've felt like my circle of friends and acquaintances was too limited.**

□ Strongly agree

□ Agree

□ Neutral

□ Disagree

□ Strongly disagree

**4. Over the past month, I have felt separated from others.**

□ Strongly agree

□ Agree

□ Neutral

□ Disagree

□ Strongly disagree

**5. Over the past month, I have felt isolated from other people**

□ Strongly agree

□ Agree

□ Neutral

□ Disagree

□ Strongly disagree

**6. For the past month, I have felt lonely and friendless.**

□ Strongly agree

□ Agree

□ Neutral

□ Disagree

□ Strongly disagree

**7. For the past month, I've felt like I had no one to rely on.**

□ Strongly agree

□ Agree

□ Neutral

□ Disagree

□ Strongly disagree

**8. Over the past month, I've thought to myself that there are enough people around me that I feel close to.**

□ Strongly agree

□ Agree

□ Neutral

□ Disagree

□ Strongly disagree

**9. Over the past month, I have felt "in tune" with the people around me.**

□ Strongly agree

□ Agree

□ Neutral

□ Disagree

□ Strongly disagree

**Ⅲ. Social network**

**The following questions are about your social network. Please mark 'v' in the appropriate box.**

**1. How many family members and relatives do you see or hear from more than once a month?**

□ None

□ 1 person

□ 2 people

□ 3-4 people

□ 5 or more people

**2. How often do you see or hear from the family members and relatives with whom you have the most contact?**

□ Once a year

□ Once every 6 months

□ 1-2 times every 3 months

□ At least once a month

□ At least once a week

**3. How many of your family and relatives do you feel comfortable enough to talk to about personal matters?**

□ None

□ 1 person

□ 2 people

□ 3-4 people

□ 5 or more people

**4. How many family members and relatives do you feel close enough to ask for help?**

□ None

□ 1 person

□ 2 people

□ 3-4 people

□ 5 or more people

**5. When one of your family members and relatives has to make an important decision, how often does he or she talk about it with you?**

□ Never

□ Rarely

□ Sometimes

□ Often

□ Very often

**6. Is there always someone you can talk to about everyday problems?**

□ None

□ 1 person

□ 2 people

□ 3-4 people

□ 5 or more people

**7. Are there a lot of people you can turn to when you have a problem?**

□ None

□ 1 person

□ 2 people

□ 3-4 people

□ 5 or more people

**8. Are there many people I can completely trust?**

□ None

□ 1 person

□ 2 people

□ 3-4 people

□ 5 or more people

**9. Do I have friends I can call whenever I need them?**

□ None

□ 1 person

□ 2 people

□ 3-4 people

□ 5 or more people

**10. How many friends do you see or hear from more than once a month?**

□ None

□ 1 person

□ 2 people

□ 3-4 people

□ 5 or more people

**11. How often do you see or hear from the friends you have the most contact with?**

□ Once a year

□ Once every 6 months

□ Once a month

□ 2-3 times a week

□ Once a day

**12. How many friends do you have with whom you feel comfortable enough to talk about personal matters?**

□ None

□ 1 person

□ 2 people

□ 3-4 people

□ 5 or more people

**13. How many friends do you feel close enough to ask for help?**

□ None

□ 1 person

□ 2 people

□ 3-4 people

□ 5 or more people

**14. When one of your friends has to make an important decision, how often does he talk about it with you?**

□ Almost never

□ Occasionally (1-2 times a week)

□ Sometimes

□ Often (5-6 times a week)

□ Always
