# Supplementary material for: Development of social isolation and social network assessment tool for older adults: A Delphi survey
Source: PLoS One. 2025 May 9;20(5):e0323198. doi: 10.1371/journal.pone.0323198 (PMC12064011; doi:10.1371/journal.pone.0323198)
Supplement: Attachment — Submitted filename: (25-0222) PONE-D-25-00848.pdf [file pone.0323198.s003.pdf]

Thank you for the opportunity to review the paper titled 'Development of Social Isolation and Social Network Assessment Tool for Older Adults: A Delphi survey'. It is a valuable agenda in an aging society, but some of the contents need to be revised as follows:

1. As the aging society accelerates and the management of the elderly is emerging as a social issue, the development of a scale that can assess social isolation and networks is a timely study. The research problem is clearly presented in the introduction.

2. In the research method section, please add exclusion criteria as well as selection criteria when recruiting an expert panel.

I think that adding theoretical background related to social isolation and networks of the elderly will further emphasize the validity of the study to the readers.

3. In the results section, please present the full names of 'SD' and 'CVR' in Tables 2 and 3 at the bottom of the table.

4. In the discussion section, I hope to add strong significance that can act as a clinical strength by using the 'Social Isolation and Network Assessment Tool for Older Adults' developed in this study. In addition, please add the limitations of the study and specifically suggest future research directions.
In addition, please more specifically present the advantages and limitations of the newly developed assessment tool compared to the existing assessment tools presented as research questions.
