# Supplementary material for: Development of social isolation and social network assessment tool for older adults: A Delphi survey
Source: PLoS One. 2025 May 9;20(5):e0323198. doi: 10.1371/journal.pone.0323198 (PMC12064011; doi:10.1371/journal.pone.0323198)
Supplement: Attachment — Submitted filename: Response to Reviewer 3.docx [file pone.0323198.s005.docx]

**Reviewer 3**

*PLOS ONE*

March 17, 2020

Dear Reviewer,

We sincerely thank you for your thorough review and valuable feedback on our manuscript. Your insightful comments and constructive suggestions have been instrumental in enhancing the quality and clarity of our work. We appreciate the time and expertise you have dedicated to evaluating our research.

We have carefully considered each of your comments and have made appropriate revisions to address your concerns. Below, we provide our point-by-point responses to your specific comments, detailing the changes implemented in the revised manuscript.

We believe that the revisions made in response to your thoughtful suggestions have substantially improved our manuscript. Your critical evaluation has helped us clarify important aspects of our research and strengthen the overall contribution of our work.

Thank you again for your valuable input and guidance throughout this review process.

Sincerely,

Park, Kang-Hyun, Ph.D. (corresponding author)

kanghyun@bu.ac.ke

- **Reviewer #3:**

1. As the aging society accelerates and the management of the elderly is emerging as a social issue, the development of a scale that can assess social isolation and networks is a timely study. The research problem is clearly presented in the introduction.

We appreciate the reviewer’s recognition of the timeliness and significance of our study. As the aging population continues to grow, addressing social isolation and networks among older adults remains a crucial issue. We are grateful for the reviewer’s positive feedback on the clarity of our research problem, which reinforces the relevance of this study.

2. In the research method section, please add exclusion criteria as well as selection criteria when recruiting an expert panel.
I think that adding theoretical background related to social isolation and networks of the elderly will further emphasize the validity of the study to the readers.

In response to the reviewer’s comment, we have added the exclusion criteria for expert panel recruitment to the Methods section to enhance transparency. Additionally, we have incorporated relevant theoretical frameworks on social isolation and networks to strengthen the study’s validity. We appreciate the reviewer’s insightful suggestions, which have improved the clarity and theoretical foundation of our research (p. 5, lines 109-111).

3. In the results section, please present the full names of ‘SD’ and ‘CVR’ in Tables 2 and 3 at the bottom of the table.

In response to the reviewer’s comment, we have added the full names of 'SD' (Standard Deviation) and 'CVR' (Content Validity Ratio) at the bottom of Tables 2 and 3 to ensure clarity. We appreciate the reviewer’s suggestion, which has improved the readability and transparency of our results section (p. 11, line 216; p. 12, line 227).

4. In the discussion section, I hope to add strong significance that can act as a clinical strength by using the ‘Social Isolation and Network Assessment Tool for Older Adults’ developed in this study. In addition, please add the limitations of the study and specifically suggest future research directions.
In addition, please more specifically present the advantages and limitations of the newly developed assessment tool compared to the existing assessment tools presented as research questions.

In response to the reviewer’s comment, we have revised the discussion section to strengthen the clinical significance of our assessment tool and provide a clearer comparison with existing tools (p. 16, lines 319-325). Additionally, we have further refined the limitations and future research directions to enhance the clarity and applicability of our findings (p. 15, lines 296-304; p. 15, lines 305-314). We appreciate the reviewer’s insightful feedback, which has helped improve the depth and impact of our discussion.
